# Comprehensive application of a systems approach to obesity prevention: a scoping review of empirical evidence

**DOI:** 10.3389/fpubh.2023.1015492

**Published:** 2023-08-08

**Authors:** Bai Li, Mohammed Alharbi, Steve Allender, Boyd Swinburn, Remco Peters, Charlie Foster

**Affiliations:** ^1^Centre for Exercise, Nutrition and Health Sciences, School for Policy Studies, University of Bristol, Bristol, United Kingdom; ^2^Global Centre for Preventive Health and Nutrition (GLOBE), Institute for Health Transformation, Faculty of Health, Deakin University, Geelong, VIC, Australia; ^3^School of Population Health, University of Auckland, Auckland, New Zealand

**Keywords:** systems approach, systems thinking, obesity prevention, group model building, system dynamics, intervention development, intervention implementation, intervention evaluation

## Abstract

A systems approach to obesity prevention is increasingly urged. However, confusion exists on what a systems approach entails in practice, and the empirical evidence on this new approach is unclear. This scoping review aimed to identify and synthesise studies/programmes that have comprehensively applied a systems approach to obesity prevention in intervention development, delivery/implementation, and evaluation. By searching international databases and grey literature, only three studies (10 publications) met inclusion criteria, which might be explained partially by suboptimal reporting. No conclusion on the effectiveness of this approach can be drawn yet due to the limited evidence base. We identified common features shared by the included studies, such as measuring ongoing changes, in addition to endpoint outcomes, and supporting capacity building. Some facilitators and barriers to applying a comprehensive systems approach in practice were identified. More well-designed and reported studies are needed, especially from low- and middle-income countries.

## 1. Introduction

Obesity is driven by interactions of complex factors, including environmental, social/cultural, political, economic, and behavioural dimensions, making obesity prevention challenging (1). Techniques from systems science have been advocated as potential tools to address this complexity (2). These tools can help identify the relationships amongst factors involved in a complex obesogenic environment/system and understand how these change over time. The use of a Causal Loop Diagram (CLD), for example, as one of the many tools, helps investigate and visualise the causal structure of a complex system, and identify feedback mechanisms and the ‘leverage points’ that produce the desired outcome(s). Previously used approaches in obesity prevention were limited in their usefulness in understanding the dynamic relationships amongst the factors that contribute to obesity. Acquiring a deeper understanding and thinking in terms of these mechanisms (feedbacks and delays), aligned with the structure and dynamics of the community, may also help design more effective and sustainable interventions to prevent obesity (3).

Multiple approaches exist to understand and address complexity within traditions of systems thinking. This means that a systems approach to tackling obesity could take different forms (4). Systems thinking approaches generally conform to ‘hard,’ ‘soft,’ or ‘critical’ traditions. Each has a particular focus within systems thinking, and has its own unique set of methods. Hard system approaches express systems in quantitative terms, and typically involve the use of mathematical modelling to predict or explain the system’s behaviour. Soft systems approaches consider the system to be an epistemological construct instead of a real-world entity. This approach involves the use of qualitative methods, and incorporates a variety of perspectives from stakeholders within the system to understand the problem (4). The critical systems tradition has its roots in the soft systems tradition though emphasises the influence and perceptions of power relations on the problem. This is perceived to be inadequately addressed in the other systems traditions (4). Despite the clear differences between the traditions, in practice these often overlap and/or work synergistically (4).

Common approaches stem from system dynamics that seek to surface and use mental models of cause and effect within specific problems and identify relationships of feedback and the impacts of change over time within a system. Any adoption of a systems approach to obesity intervention should be informed by a clearly defined branch of systems sciences. Approaches should recognise nonlinear and dynamic interactions between variables operating across different levels or subsystems within the environment where a target population lives. Intervention development, implementation and evaluation must actively engage with this complexity both across and within intervention components/settings. This means that an intervention which solely comprises multiple components and/or operating at multiple settings is not necessarily an intervention taking a systems approach (5–7).

Applying a systems approach involves utilising mental/computational models, feedback loops and structures within a system; and this may re-orient the goals, structures, and resources of the system (5, 7). Models are formed based on the scientific and/or practical knowledge of the people who have built them. They provide a visual presentation of the system or problem being investigated. Feedback loops which can be reinforcing and balancing, describe cause and effect relationships.

Despite the concepts and terminology of systems approaches existing for several decades (8, 9), empirical knowledge about their application and effectiveness for obesity prevention is limited. More clarity is required regarding what systems-based obesity prevention interventions look like in practice.

Several reviews have used the term ‘whole system approach’ (WSA) to identify obesity prevention programmes. In 2010–2011, three reviews were conducted by the National Institute for Health and Care Excellence (NICE) to identify the key elements (6), effectiveness (10) and barriers/facilitators (11) of WSA to obesity prevention. However, due to lack of studies, these NICE reviews widened the definition of WSA to include multi-level/multi-setting programmes and proposed a list of 10 features of a WSA to tackle obesity based on their wider definition. A later systematic review aimed to synthesise available evidence on WSA targeting obesity and other public health areas based on the NICE 10 proposed WSA features (12). However, since these features were developed based on studies that did not show all characteristics of a systems approach, this later review included multi-level/multi-setting interventions. The authors of this review recognised the need to re-define WSA. The 2019 Public Health England guide to support local approaches to promoting a healthy weight using a WSA offered a better description of WSA (13). Although some of the case studies included in this guidance might not show evidence of taking a systems approach in all intervention stages, the definition and guidance offered in this document recognise essential features of systems thinking. Thus, they are helpful for the academic community, public health practitioners and policy makers in a practical sense. More recently, a systematic review identified different systems methods used to evaluate public health interventions.

To date, no systematic reviews have been specifically designed to identify programmes or studies that applied systems thinking across all stages of an intervention’s life cycle.

Therefore, we conducted a systematic scoping review to identify and synthesise programmes/studies that have comprehensively used a systems approach to address obesity. A ‘comprehensive’ application of a systems approach should demonstrate systems thinking in all key stages of an intervention’s life cycle, not just at the development stage. As a result, we excluded some studies that applied a systems approach at the intervention development stage but did not clearly report how they implemented or evaluated the developed interventions in a way that demonstrated systems thinking. It is important to note that the purpose of our review was not to identify or define authentic applications of a systems approach to obesity interventions.

1. Our specific research questions were: How many studies or intervention programmes have made a comprehensive application of a systems approach to obesity prevention? (see Methods for our inclusion criteria)?
2. What is the available empirical evidence on the effectiveness of included programmes/studies that demonstrated systems thinking in all stages of the intervention’s life cycle?
3. Were there any adaptations incorporated into the systems approach to obesity prevention to suit different settings?
4. What were the main features shared by studies/programmes that made a comprehensive application of a systems approach to obesity prevention?
5. What are the reported barriers and facilitators to applying this systems approach to obesity prevention?

## 2. Methods

Our review adopted the five stages framework provided by Arksey and O’Malley (14) and Levac et al. (15) and used the reporting criteria of The PRISMA Extension for Scoping Reviews (PRISMA-ScR) (16). For research question 2, studies should have reported at least behavioural or anthropometric outcomes. In addition, we included other outcomes, such as intervention implementation, cost-effectiveness, and psychosocial impact. Any peer-reviewed research or grey literature was considered. We excluded theoretical literature, editorials, opinion pieces/commentaries and conference abstracts. We also excluded studies that used systems science to understand the mechanisms of obesity unless these aimed to inform the development of a systems-based intervention and the intervention has been implemented/evaluated. To be considered a **comprehensive application of a systems approach**, studies/programmes had to meet all the following criteria associated with the development, delivery/implementation, and monitoring/evaluation stages of an intervention’s life cycle:

- The process to develop the intervention featured all the principle steps for transformative systems change provided by the Foster-Fishman’s framework (17) (Table 1).
- The chosen approach to deliver (for experimental purpose) or implement (as a public health initiative) the intervention showed evidence of recognising the dynamic and complex nature of the intervention and the system for which the intervention was developed.
- The chosen approach to monitor/evaluate the developed intervention also showed evidence of recognising the dynamic and complex nature of the intervention and the system for which the intervention was developed.

**Table 1:** Foster-Fishman framework (16).

Bounding the system	Understanding system parts as root causes	Assessing system interactions	Identifying levers for change
Problem definition Identification of the levels, niches, organisations, and actors relevant to the problem	System norms, resources, regulations, operations.	Reinforcing and balancing interdependencies System feedback and self-regulation Interaction delays	**Identifying parts to leverage for change:** Exerts or could exert cross-level influences Directs system behaviour Feasible to change **Identifying interactions and patterns to leverage for change:** System differences that create niches compatible with systems change goals Long-standing patterns that support or hinder change goals Gaps in system feedback mechanisms Cross-level/sector connections that are needed.

The Foster-Fishman’s framework was selected as a part of our criteria during the study selection process. The framework provides some clarity about what a systems approach to intervention development might entail. It describes systems approaches as comprising ‘bounding the system,’ ‘understanding system parts as root causes,’ ‘assessing system interactions,’ and ‘identifying levers for change’ (17).

Several questions were used to determine study eligibility against each intervention stage. For example, for the development stage, we considered ‘have the authors specified the theoretical underpinning of the systems approach applied to develop the intervention and justified their choice?’; and ‘have the authors described clearly the methods applied to develop the intervention and justified their choice?’

For the implantation stage, example questions were: ‘have the authors specified the responsibilities of all individuals and organisations involved in the delivery of jointly identified and prioritised intervention actions?’; and ‘have the authors described in sufficient detail what were delivered/implemented, including the initial plan and subsequent changes to the initial plan?.’ For the evaluation stage, we asked, for example, ‘have any evaluation outcomes been used to review and update stakeholders’ understanding of the system gained collectively prior to intervention delivery?’

We did not apply any restrictions on research/community settings or participants characteristics. We searched the following databases from inception to February 2021: Web of Science, PubMed, and MEDLINE. Moreover, grey literature was searched with particular attention to significant bodies, and hand searches were also used. Search terms are provided in Supplementary material 1.

We imported all references and removed duplicates in Covidence online software (18). Two reviewers independently conducted the titles and abstracts screening and selected articles based on the predetermined inclusion and exclusion criteria. Then, we extracted and recorded relevant data using a customised form. We extracted data on the author(s), year and type of publication, location/setting, targeted participants or population group, study aims, systems methods/tools, intervention details, study design, outcome measures, and key findings from each programme/study. The Consolidated Standards of Reporting Trials (CONSORT) extension abstracts (SW-CRT) (19) and the standard Critical Appraisal Skills Programme and EPPI-Centre tools (20) were used to assess the included studies.

## 3. Results

### 3.1. Articles retrieved

**Figure 1:**
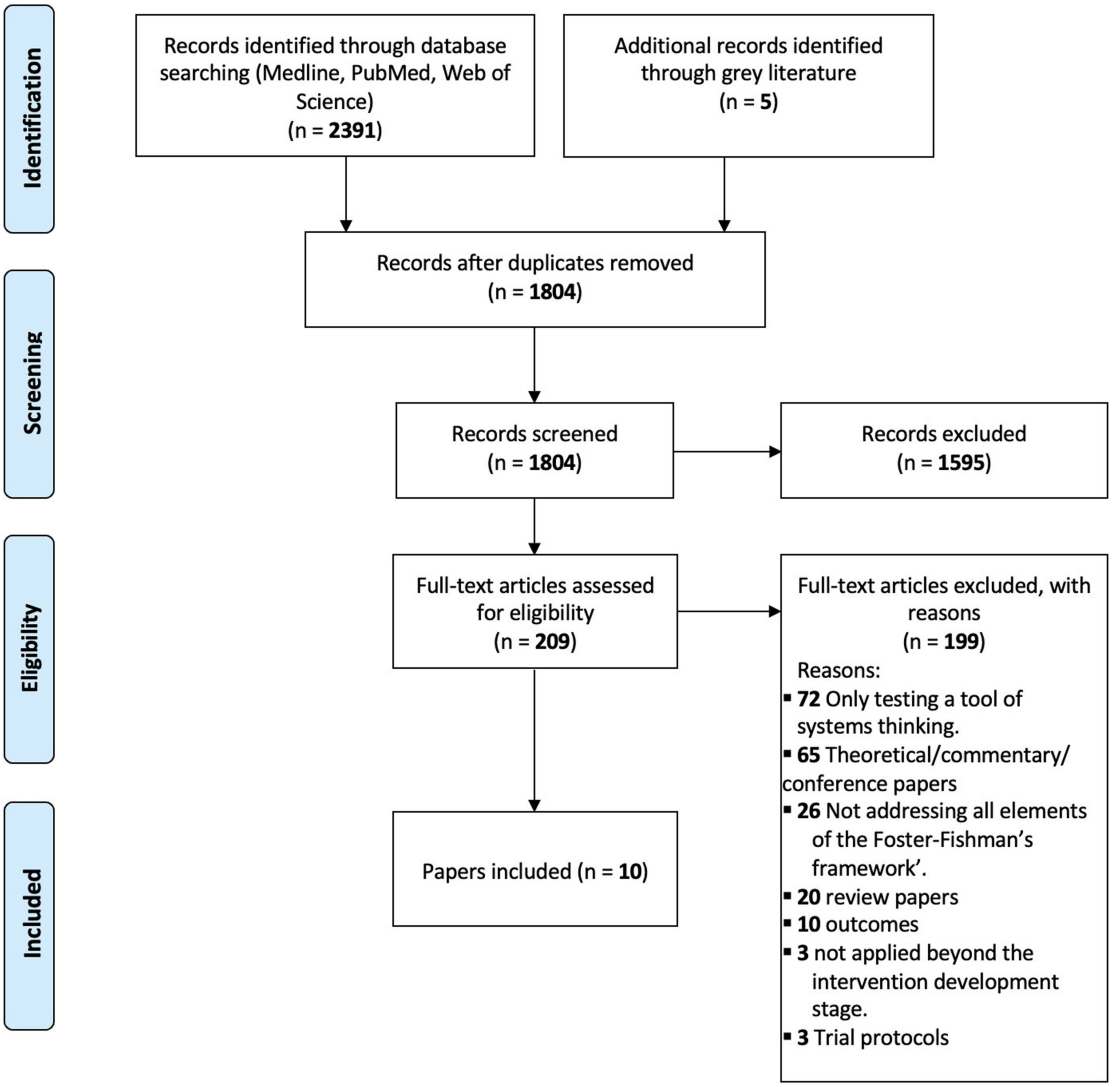
Flow diagram of scoping review study identification.

We identified 2,396 articles. After removing duplicates, 1,804 records underwent title and abstract screening, and 209 underwent full-text review (Figure 1). Of these, 10 articles met the inclusion criteria.

Ten articles from two countries were published between 2016 and 2022. Three articles describe the design and methods of three programmes for preventing childhood obesity (21–23). The remaining seven articles report on a process evaluation and the key findings of the included intervention programmes (24–30).

### 3.2. How many studies or intervention programmes have made a comprehensive application of a systems approach to obesity prevention according to the definition used in this review?

Three obesity prevention interventions meet our inclusion criteria to apply a systems approach to obesity prevention (Table 2) comprehensively. All excluded studies/programmes and reasons for exclusion are provided in Supplementary material 2. We describe the three included programmes below.

**Table 2:** A summary of studies that made a comprehensive application of a systems approach to developing, delivering and evaluating childhood obesity prevention according to the criteria used in this review.

Study title and years	Country	Target Population	Programme/Intervention characteristics^a^	Evaluation approach	Outcomes	Systems method and Theoretical basis	Trial findings/status	Comprehensiveness as defined in this review
WHO STOPS Childhood Obesity (20, 24)2016–2021	Australia	Children (grade 2, 4, 6; ages 7–12 years)	Assessing alignment of current system with values and assumptions of the targeted outcome. Exploring the interactions and interdependencies amongst system subsystems or components Using the established systems map to identify intervention opportunities and convert these to community-built and systems-oriented action plans.	A stepped-wedged cluster randomised controlled trial	Anthropometry (BMIz^b^, overweight/obesity prevalence) Physical activity and sedentary behaviour Diet type, frequency Quality of life Environments Social networks (ecological data) Process/implementation indicators	Group Model Building (GMB), Systems dynamics	Trial findings have been published.**Main Result:***WHOSTOPS* had a positive impact on takeaway food intake and health-related quality of life.A full summary was included in section 3.4.	A systems approach was used in all stages of the intervention’s life cycle (intervention development, delivery and evaluation)
RESPOND (22)2018–2023	Australia	birth to 12 years	**Five components:**1. Systems approach capacity building for each participating community: *Face-to-face GMB training to develop local interventions* *Online support* 2. Community-led intervention activity.3. School Monitoring System and analysis of weight status of infants and young children aged 2 and 3.5 years (via de-identified Maternal and Child Health Data)4. Knowledge, engagement and social network analyses (using surveys to collect data on changes over time relevant to obesity amongst children)5. Collaborative Governance and Implementation Structure (Collective Impact).	A stepped-wedged cluster randomised controlled trial	BMIz^b^ Overweight/obesity prevalence Typical/usual serves of non-core (discretionary) foods consumed daily	Group Model Building (GMB), Systems dynamics	Data from the baseline measurement has been presented.The study has entered step two of the stepped wedge trial design	A systems approach was used in all stages of the intervention’s life cycle (development, delivery, and evaluation)
The LIKE (21)2018–2022	Netherlands	10–14 years	Understanding the pre-existing systems about dietary, physical activity, sleep, and screen-based sedentary behaviours. Summarising findings in a systems map and using Social Network Analysis (SNA) to identify the influential actors (societal stakeholders/policymakers) to develop actions using GMB workshops. Use the understanding gleaned in the systems maps to support adaptation, ongoing programme development, and feedback on broad systems change as the intervention programme was implemented.	Developmental systems evaluation, supplemented with routinely collected data	BMIz^b^ Behavioural (diet, physical activity, screen use and sleep)	Group Model Building (GMB), social network analysis (SNA), Systems dynamics	The study has been running since 2017 and will be completed (including a dynamic evaluation) in July 2023	A systems approach was used in all stages of the intervention’s life cycle (intervention development, delivery and evaluation)

a Unlike traditional interventions studies where interventions components are clearly defined and adherent to protocol, the interventions implemented or delivered in the three included studies involved GMB and capacity building work in participating communities. The intervention actions varied between communities and evolved (were modified) in response to ongoing evaluation results. This is an important sign of the comprehensive application of systems approach to obesity prevention.

b Body mass index z score (standard deviation score).

#### 3.2.1. The whole of systems trial of prevention strategies for childhood obesity and the reflexive evidence and systems interventions to prevent obesity and non-communicable disease study

These two studies were conducted in Victoria, Australia, using a similar methodological approach (systems dynamic) to intervention development, implementation, and evaluation (21). The whole of systems trial of prevention strategies for childhood obesity (WHOSTOPS) study (Western Victoria) predated reflexive evidence and systems interventions to prevent obesity and non-communicable disease (RESPOND; Northern Victoria), the latter extended the approach pioneered in the earlier trial (23).

##### 3.2.1.1. Intervention development

Both studies (21, 23, 28–30) facilitated a deeper and shared understanding of system components such as systems norms, human resources, social resources, economic resources, operations, and regulations. This included assessing alignment of current system with values and assumptions of targeted outcome or change and assessing degree to which current system has in place or is building the infrastructure to support goals or targeted outcome.

In the next step, both studies started to explore the interactions and interdependencies amongst system subsystems or components and how the system self-regulates using Group Model Building (GMB) workshops and co-produced CLD. GMB is a system dynamics method that provides a workshop structure to engage diverse stakeholders in collective activities to create a dynamic system model known as a CLD. These visualised the nonlinear and dynamic interactions between variables operating across different levels or subsystems within the environment. The CLD was used as a representation of the system at the third workshop with a broad group of community members to identify and prioritise levers that can be used to change the system (21, 23, 28–30).

##### 3.2.1.2. Intervention implementation

Each intervention was oriented around strengthening leadership, workforce development, resources, partners, networks and intelligence through intensive training and support for each intervention community. The system intervention was carried out with community stakeholders who had authority or control over the environments in which children were exposed to the identified system drivers risk factors. For WHSTOPS, the research team delivered the GMB workshops and were actively involved in planning implementation. In contrast, for RESPOND, the research team trained local community and health staff to deliver this process, plus a new and existing coalition of community leaders was convened to lead community-wide structural change (21, 23, 28–30). Both studies formed a steering group to prioritise changing systems to support physical activity, healthier food choices and childhood obesity prevention across the intervention design process.

##### 3.2.1.3. Intervention evaluation

Both studies used a stepped-wedged randomised control trial design (SW RCT) to minimise practical and ethical issues associated with complex, population-level interventions (25, 28–30). Stepped-wedged randomised control trial is one of the recommended study designs for evaluating complex interventions that involve whole-community policy/service changes that require political, logistic, and ethical consideration (31). Moreover, the WHOSTOPS evaluation approach included continuous outcome measurement (vs. measuring outcomes at certain endpoints). This showed recognition of the dynamic nature of implemented interventions, and continuous data collection made investigation of how system changes occurred possible’.

#### 3.2.2. Lifestyle innovations based youth’s knowledge and experience (the LIKE programme)

LIKE was a 5-year study set in three districts in Amsterdam, with an intended overrepresentation of lower socio-economic and ethnic minority groups (22). It aimed to build a dynamic action programme based on the current system. It evaluated (1) how the system evolved in response to the developed programme and (2) how it contributed to improvements in health-related behaviours and prevalence of overweight and obesity amongst children aged 10 to 14 years old.

##### 3.2.2.1. Intervention development

The LIKE programme was started by understanding the pre-existing systems that contribute to determinants of dietary, physical activity, sleep, and screen-based sedentary behaviours in the target population (22, 26, 27). Findings related to these determinants were summarised in a systems map built using GMB. This map was used as a reference for developing actions and as a basis for evaluation. They used Social Network Analysis (SNA) to identify the influential actors who hold a central position within the local governance and/or at community level and invited them to develop actions through the use of GMB workshops (22, 26, 27).

##### 3.2.2.2. Intervention delivery and evaluation

The evaluation used developmental systems approaches, supplemented with routinely collected data on weight status and key health behavioural indicators (22, 26, 27). A key stated aspect of this approach was using the understanding gleaned in the systems maps to support adaptation, ongoing programme development, and feedback on broad systems change as the intervention programme was implemented. In other words, the intervention was being developed, implemented, monitored and re-developed in a continuous, adaptive process (22, 26, 27).

### 3.3. What is the available empirical evidence on the effectiveness of this intervention approach?

Only WHOSTOPS paper (25) reported the effectiveness of using a comprehensive systems approach to obesity prevention. No effectiveness findings had been reported for other included interventions at the time of writing.

WHOSTOPS was evaluated using a SW-RCT design over 4 years and reported a significant decline in mean BMI z score in the intervention group within the first 2 years followed by an increase. The mean BMI z score amongst the control group remained unchanged throughout the study period (25). A similar ‘U shape’ pattern of change was observed for the percentage of overweight/obesity in the intervention group, whilst the corresponding figure for the control group remained stable. There was an intervention by time interaction in BMI z scores (*p* = 0·031). The authors suggested several contextual explanations for such findings. First, as planned, the research team reduced their implementation support to step-one communities in the second year to focus more on recruiting communities for step two. Due to bushfires and other natural disasters, control communities had to delay intervention uptake for 2 years. The resources allocated to the first set of intervention communities was reduced by at least half of what was planned for the last 2 years of the study. Second, there might be an unintended consequence (e.g., complacency, a feeling of the job being done and shifting priorities) of seeing early signs of a positive outcome in the intervention communities. The study did not achieve the desired sample size of 1,500 in each trial arm and was underpowered to detect hypothesised BMI z score change (25).

### 3.4. Were there any adaptations incorporated into the systems approach to suit different research settings?

No adaptions were reported for the included programmes. The WHOSTOPS, RESPOND and LIKE (21–23) were each developed using GMB. In each case, these methods were underpinned by previously developed scripts to design and run these sessions. The scripts themselves provide scope for the design team to adapt the framing of the question, the scale of the target area and the systems requiring attention.

### 3.5. What were the main features shared by studies that have made a comprehensive application of a systems approach to obesity prevention?

The main features shared by all three included studies (21–23) are described below.

#### 3.5.1. Mapping the systems of obesity drivers and embedding actions within the systems

The WHOSTOPS, RESPOND and LIKE (21–23) used a systems lens to understand the various system levels and interventions required for sustainable, large-scale changes. GMB workshops as a systems dynamic tool were used in all studies to create a system map that recognises nonlinear and dynamic interactions between variables operating across different levels or subsystems within the target population’s environment. All programmes (1) started with understanding current systems and contexts within the communities; (2) identified, prioritised, and acted on systemic drivers of obesity; and (3) identified ways in which current systems and resources can be re-oriented or used for better health outcomes. All three studies used the *Systems Thinking for Community Knowledge Exchange* (STICKE) software to support the process. STICKE was initially developed to support WHOSTOPS (32) and subsequently is continually adapted to meet the needs of the communities in terms of increasing understanding and aligning with their existing planning and reporting requirements (33).

#### 3.5.2. Measuring ongoing changes not just the endpoint outcomes

All studies (21–23) demonstrated systems thinking throughout the development, implementation, and evaluation stages of their intervention’s life cycle. Most notably, at the evaluation stage, all studies included evaluation and tracking of changes in the systems (34). Such an evaluation and monitoring approach is necessary given the dynamic and adaptive nature of any system. For example, within the WHOSTOPS study (21), ongoing data collection and updates of the systems map helped to optimise implementation and facilitate diffusion of the selected actions; new ideas were stimulated in an adaptive, constructive, capacity-building cycle. In depth interviews with community practitioners demonstrated how data helped frame the priorities of community prevention efforts to child health behaviours and the continual mapping process helped leaders to identify and track junk food, physical inactivity and moves from programmatic approaches as key areas of focus (28).

#### 3.5.3. Measuring intervention processes

All studies undertook a process evaluation to understand how successfully the systems approach created a sustainable programme and how communities responded to systems interventions. Just as with ongoing outcome measurements, process evaluation can also inform adaptive/new actions to optimise intervention outcomes. Both the knowledge about and interventions on the systems are advanced continuously. However, no authors reported whether or how process evaluation contributed to learning how the systems worked.

#### 3.5.4. Local decision-makers and influential actors lead and own intervention development and implementation

A common feature across studies (21–23) was that researchers in these studies supported local decision-makers and influential actors to develop and implement systemic interventions for transformative systems change through a co-creation, participatory approach. Those individuals were leaders from local government and other key sectors/subsystems of the communities (21–23). They have the authority, power, and/or resources to approve and/or implement prioritised interventions. In the WHOSTOPS and RESPOND studies, community leaders who directly affected pre-adolescent environments were invited to develop and implement interventions (21, 23). Social Network Analysis was used in LIKE to identify influential actors who were then invited to participate in all parts of the project (22).

#### 3.5.5. Supporting capacity building as an essential goal alongside achieving clinical effectiveness

All included studies have explicitly spent effort to strengthen the World Health Organisation (WHO) system building blocks (35, 37), including leadership, resources, partnership and intelligence in community settings. For example, the WHOSTOPS study convened a new and existing coalition of community leaders who have the capacity and network to lead systems change across the community. The strength and structure of this network and influence on action is reported in relation to the initial system map developed by the community (29). Moreover, the RESPOND study trained local community leaders to run GMB workshops. One result of this capacity building is the use of techniques in these communities for problems outside the initial intent to address to childhood obesity (21, 23). For example, several RESPOND communities used GMB and systems methods to understand and plan responses to food insecurity arising from the COVID-19 pandemic (30). Furthermore, the LIKE study invited adolescents to a capacity building workshop to teach them how to conduct research amongst their peers about healthy behaviours and potential actions towards stimulating healthy behaviours.

### 3.6. What are the reported facilitators and barriers to applying a systems approach to obesity prevention identified by the included studies?

Only one article (24) reported barriers and enablers. This article is a process evaluation of a pilot community that participated in the WHOSTOPS (21) programme in Victoria, Australia’s Great South Coast region.

The GMB workshops and ‘the organic evolution’ of the programme in all areas and levels of the system were reported by the steering and community task team members to be helpful. This approach established community ownership of the system by engaging a diverse range of community members who collectively unpacked the complexity of obesity and its main influences (24). Furthermore, co-creation teamwork, including sharing information within the steering group, engaging local agencies, and commitment of authorities to integrated working, has been identified to positively impact the programme’s feeling of ownership, development, and progression (24).

Focusing on community assets rather than needs or lacks was helpful in information sharing between members, engaging relevant organisations, forming a relationship with a topic expert, and attaining the commitment of many local authorities to participate in the collaboration (24). This can be accomplished by shifting mindsets from deficits to capabilities, highlighting and connecting a varied range of community assets and mobilising the connected assets for action (38).

Triggers to personal involvement in the programme and perceived prompts for others to participate have been identified as important facilitators of engagement in the process. For instance, the use of GMB has been found as a powerful tool to promote a shared understanding of the complexities of obesity in the local context and the need for collective actions (24).

Some of the identified barriers are miscommunication and confusion observed within the steering group organisation regarding individual responsibilities and roles. As a result, thought processes amongst members of the steering groups were not always aligned. Furthermore, a lack of support to those working at a lower level was identified within the steering group (24). Another barrier is related to the lack of application of the asset-based community development (ABCD) approach that promotes ownership and sustainability and could have been more effective if it occurred in conjunction with the GMB workshop (24).

The standard processes of GMB workshops were not adapted to support community members who had low health literacy, and no additional efforts were undertaken (24). This may negatively affect the efficiency of the task teams. Another identified barrier is related to unforeseen social and economic shocks. For WHOSTOPS, the bushfire impacted the subsequent delivery of intervention (25), which will be even more marked when we understand the impact of COVID.

### 3.7. Quality assessment

The quality of two papers (24, 25) was assessed by an appropriate tool based on their study designs. We only assessed these two papers since these reported interventions outcomes. The WHOSTOPS met 14 of 17 of the reporting quality items of the Consolidated Standards of Reporting Trials (CONSORT) extension for the stepped wedge cluster randomised trial (SW-CRT; see in Supplementary material 3). The process evaluation study (24) was assessed using the SCAS-EPPI (20). The reliability of the included process evaluation findings was rated as a medium, whilst the usefulness of the findings was rated as high (see in Supplementary material 4).

## 4. Discussion

This review included 10 publications (21–30) reporting on three eligible studies (21–23). This number suggests that comprehensive application of a systems approach to obesity prevention is limited. Although there is positive evidence, more empirical evidence is needed to understand the application and effectiveness of this approach. Furthermore, no empirical evidence is available from non-western, developing settings.

The scarcity of studies using a comprehensive systems approach may partly be due to the uncertainty around the exact meaning of ‘a systems approach’. Some programmes appeared to implement multi-level, multi-component interventions, or did not meet our inclusion criteria for intervention development (Supplementary material 2). Moreover, sub-optimal reporting might have also explained the small number of studies meeting our inclusion criteria. The 2019 systematic review also found that the reporting of most included studies lacked sufficient detail (12). Similarly, authors of the recent review on different methods used to evaluate various public health interventions also suggested that more consideration could be given on how to present findings from complex systems evaluation (36). Therefore, robust and well-reported evidence is needed to improve our understanding of how a systems approach can be applied practically. To address this issue, we developed a practical guidance for reporting health interventions underpinned by a systems approach (39). This guidance is presented in a format of practical questions to assist academic authors, journal editors and other interested stakeholders to design, report or review future interventions that apply a systems approach to tackle obesity or other public health challenges. These questions were developed based on our empirical experience of applying a systems approach to health promotion across 16 countries, and comparative reflections on what were reported by studies included in this review and what were not reported by excluded but potentially eligible studies (those that were excluded due to insufficient reporting). The guiding questions are organised by the three interrelated stages of an intervention’s life cycle: ‘development’ (10 guiding questions), ‘implementation/delivery’ (10 guiding questions) and ‘evaluation/monitoring’ (12 guiding questions).

Our review only found one article that reported on the effectiveness of the WHOSTOPS programme. Therefore, published evidence on the impact of taking a comprehensive approach to obesity prevention is still limited. However, we are aware of several ongoing studies that will publish their evaluation outcomes within the next few years. Overall, WHOSTOPS was found to positively impact health-related quality of life, take-away consumption and water consumption amongst girls, and packaged snacks amongst boys (25). However, a ‘U shaped’ pattern was observed for changes in mean BMI z-scores and overweight/obesity percentages amongst the intervention communities, whilst these two outcomes remained largely unchanged amongst the comparison communities throughout the study period. A valuable finding from this study was the suggested explanation (explained in section 3.4) for such findings by the programme’s/study’s researchers. Furthermore, the length of an intervention might be critical in determining measured intervention outcomes. A systematic review of 26 obesity prevention studies focused on the same age group (7–12 years) as WHOSTOPS found that interventions lasting 12 months or less were most effective in preventing obesity (40). Future research should pay attention to potential interactions between intervention length and impact.

Our review did not limit searches to English-language publications only but all included studies (21–23) were based in western, high-income countries (Australia and the Netherlands). Although it is possible that eligible research that is not archived by international databases might have been missed, we believe this is unlikely given the origin and early stage of applying systems approaches to obesity interventions. This finding raises an important question about the feasibility of applying a systems approach in non-western and/or developing countries. One challenge might be realising cross-boundary collaboration amongst authorities and organisations to tackle health issues. For example, a study conducted in a Middle East country found that collaboration amongst diverse stakeholders is limited due to cultural and gender barriers (41). Moreover, many non-western countries adopted a highly centralised governing model in which the central authority has more strict control over local authorities. This could be a particular challenge when implementing a systems approach to public health intervention development and implementation as this approach is bottom-up and collaborative. Moreover, a centralised government can disempower local councils and not view health promotion or disease prevention activities as politically favourable (42, 43). These challenges imply that the feasibility of using the systems approach in non-western countries should be a focus of future research.

Our review identified common features shared by studies that were considered to have comprehensively applied a systems approach to obesity prevention. Similarly, the 2019 review (12) and the NICE review (8) found that building relationships and community capacity was required to create successful outcomes.

Our review identified only one process evaluation (24) of an included intervention. This makes it challenging to provide a comprehensive summary of reported barriers and facilitators to applying a systems approach to obesity prevention. However, the identified barriers and facilitators can improve the design and delivery of future obesity interventions that take a comprehensive systems approach. For example, focusing on community assets will create a complete picture of shared motivations for change. This increases the possibility that change efforts will receive widespread support and success (38). Moreover, a strong reciprocal relationship was identified between systems thinking, collective impact and asset-based community development. Using these concepts seems to prevent an intervention programme (at least in the short term) from reverting back to business as usual (24, 44–46).

This is the first review to identify and assess published evidence of a systems approach to obesity prevention using strict inclusion criteria to encompass all stages of an intervention’s life cycle. This is the main strength of our review since previous reviews applied broader inclusion criteria. A wide range of data sources, outcomes and process evaluation were included to capture all available evidence. Moreover, common features of comprehensive use of a systems approach to obesity prevention and application facilitators and barriers were identified.

The review also has limitations. First, there are two sides to applying strict inclusion criteria in this review. Although strict inclusion criteria allowed us to identify and synthesise evidence from studies that applied a systems approach at all stages of the intervention life cycle, some valuable knowledge generated by studies that only met our inclusion criteria partially was not captured by this review. Second, our definition of comprehensive use of a systems approach to obesity prevention was determined based on the current academic knowledge and our empirical experience. Our definition and review may be updated accordingly as the practical application of a systems approach to obesity prevention, and other public health challenges are advanced. Moreover, it is possible that some studies/programmes might have made comprehensive use of a systems approach but were excluded from this review for lacking methodological and process details in associated publications. This might mean that findings on other eligible studies/programmes were not considered in this review. There is an urgent need to develop practical guidance for reporting public health interventions underpinned by a systems approach to advance evidence synthesis and methodological development. Furthermore, we identified evidence for the effectiveness of this approach on behavioural outcomes and quality of life. However, this was based on one included study. More research is needed to understand better the impact of adopting a comprehensive systems approach to obesity prevention. Researchers and authors should also report major changes in the intervention environment and reflect on how such changes might have influenced intervention outcomes at different times. Non-western researchers are encouraged to test the approach in their settings and report any culturally relevant adaptations made to existing processes and tools.

## 5. Conclusion

Our review identified only three studies considered to have made a comprehensive application of a systems approach to obesity prevention intervention. This might be due to a misunderstanding of this approach or insufficient reporting of key processes and methods. Currently, no published empirical evidence is available from outside western, high-income settings. The evidence for the effectiveness of this approach on behavioural outcomes and quality of life was identified based on one included study. However, given this extremely limited evidence base, no conclusion on the effectiveness of this approach can be drawn yet. This review also identified common features shared by included studies, which may help clarify existing confusions around the meaning and practical application of a systems approach to obesity prevention. Finally, some barriers and facilitators to applying a comprehensive systems approach in practice were identified, and they would help improve the design and implementation of future work.

## Data availability statement

The original contributions presented in the study are included in the article/Supplementary material, further inquiries can be directed to the corresponding authors.

## Author contributions

BL conceived the study idea and led the development of the study design. CF provided methodological advice and supervised the study with BL. MA conducted the literature search, screening/selection of papers, and data extraction and analysis. SA provided training and theoretical and methodological advice. BS provided theoretical expertise. RP worked as a second reviewer during the screening and selection of papers. Any disagreements between MA and RP over the eligibility of specific studies, the data extraction process and the quality assessment process were resolved by discussion with BL. BL, MA, SA, BS, RP, and CF contributed to the interpretation of the review findings. MA drafted the manuscript, which was revised substantially by BL, CF, and SA. All authors contributed to the article and approved the submitted version.

## Funding

This paper was an output from the SYSTAM CHINA SEACS project funded by the UK Medical Research Council (grant number: MR/V004174/1).

## Conflict of interest

The authors declare that the research was conducted in the absence of any commercial or financial relationships that could be construed as a potential conflict of interest.

## Publisher’s note

All claims expressed in this article are solely those of the authors and do not necessarily represent those of their affiliated organizations, or those of the publisher, the editors and the reviewers. Any product that may be evaluated in this article, or claim that may be made by its manufacturer, is not guaranteed or endorsed by the publisher.
