# Supplementary material for: Comprehensive application of a systems approach to obesity prevention: a scoping review of empirical evidence
Source: Front Public Health. 2023 Aug 8;11:1015492. doi: 10.3389/fpubh.2023.1015492 (PMC10442543; doi:10.3389/fpubh.2023.1015492)
Supplement: Supplementary file 1 [file Data_Sheet_1.docx]

**Supplementary 1: List of terms used in the search strategy**

Whole systems approach

System*-based approach

System* science

System* dynamics

System* approach

System* interventions

Dynamic simulation model*

System* thinking

Dynamic behavi*

System* modelling

Agent-based model*

Group model building

Social network analysis

Nonlinear dynamics

Computer simulation

Systems analysis

Overweight

Bodyweight

Obesity

Weight gain

Paediatric obesity

Adiposity

Body mass index

**Supplementary 2: A list of excluded programmes and reasons for exclusion.**

|  | **Study title** | **Main author and year** | **Reason for exclusion** |
| --- | --- | --- | --- |
|  | A rural community moves closer to sustainable obesity prevention - an exploration of community readiness pre and post a community-based participatory intervention | Whelan, j. 2019 | Did not provide details on how the programme developed. |
|  | Projected incidence of overweight and obesity and related disease incidence across poland | Webber 2014 | Theoretical or commentary papers |
|  | Understanding obesity-related behaviors in youth from a systems dynamics perspective: the use of causal loop diagrams | Waterlander 2020 | Only testing a tool of systems thinking |
|  | Applications of systems science in biomedical research regarding obesity and noncommunicable chronic diseases: opportunities, promise, and challenges | Wang, y. 2015 | Theoretical or commentary papers |
|  | Applications of complex systems science in obesity and noncommunicable chronic disease research | Wang, y. F. 2014 | Theoretical or commentary papers |
|  | Food futures: developing effective food systems interventions to improve public health nutrition | Waterlander, w. 2018 | Theoretical or commentary papers |
|  | Exploring the dynamics of food-related policymaking processes and evidence use in fiji using systems thinking | Waqa 2017 | Only testing a tool of systems thinking |
|  | Factors affecting evidence-use in food policy-making processes in health and agriculture in fiji | Waqa 2017 | Only testing a tool of systems thinking |
|  | The effect of food portion sizes on the obesity prevention using system dynamics modelling | Abidin 2014 | Only testing a tool of systems thinking |
|  | Combating obesity through healthy eating behavior: a call for system dynamics optimization | Abidin 2014 | Theoretical or commentary papers |
|  | Examining social norm impacts on obesity and eating behaviors among US school children based on agent-based model | Wang 2014 | Only testing a tool of systems thinking |
|  | Simulation analysis on the consequences of behavioural change towards combating obesity: system dynamics approach | Abidin 2016 | Theoretical or commentary papers |
|  | A system dynamics optimization framework to achieve population desired of average weight target | Abidin 2017 | Theoretical or commentary papers |
|  | Modeling the effect of sedentary behaviour on the prevention of population obesity using the system dynamics approach | Abidin 2015 | Only testing a tool of systems thinking |
|  | The global effect of childhood obesity a whole systems approach is needed | Wall 2017 | Theoretical or commentary papers |
|  | Identifying influence agents that promote physical activity through the simulation of social network interventions: agent-based modeling study | Vanwoudenberg 2019 | Only testing a tool of systems thinking |
|  | Building health communities: local health care networks in maryland | Wachhaus 2020 | Only testing a tool of systems thinking |
|  | A complex system approach to address world challenges in food and agriculture | Vanmil 2014 | Theoretical or commentary papers |
|  | Community-based pilot intervention to tackle childhood obesity: a whole-system approach | Vamos 2016 | The programme did not use any tools of systems thinking, and didn’t bound the system, interventions were not based on interactions of interrelated factors. |
|  | Improving stability of prediction models based on correlated omics data by using network approaches | Tissier 2018 | Theoretical or commentary papers |
|  | Cost-effectiveness of population-based, community, workplace 24and individual policies for diabetes prevention in the uk | Breeze 2017 | Outcomes |
|  | Identifying the effects of environ27mental and policy change in28terventions on healthy eating | Bowen 2015 | Review papers |
|  | Commentary: pediatric obesity: systems science strategies for prevention | Black 2013 | Theoretical or commentary papers |
|  | A systems approach to reducing maternal obesity: the health in preconception, pregnancy and postbirth (hippp) collaborative | Skouteris 2015 | Theoretical or commentary papers |
|  | Economic evaluations of system-based obesity interventions - the case for a new approach | Sonntag 2018 | Theoretical or commentary papers |
|  | Community readiness assessment for obesity research: pilot implementation of the healthier families programme | Teeters 2018 | Assessing the readiness before developing a systems approach interventions |
|  | Group model building: a participatory approach to understanding and acting on systems | Siokou 2014 | Theoretical or commentary papers |
|  | The dynamic relationship between unhealthy weight control and adolescent friendships: a social network approach | Simone 2018 | Only testing a tool of systems thinking |
|  | Social network analysis of adolescent obesity | Shoham 2009 | Theoretical or commentary papers |
|  | The outcomes of health-promoting communities: being active eating well initiative-a community-based obesity prevention intervention in victoria, australia | Bolton 2017 | Using multi components interventions, did not bound the system, did not use mental models |
|  | Protocol for the measurement of changes in knowledge and engagement in the stepped wedge cluster randomised trial for childhood obesity prevention in australia: (reflexive evidence and systems interventions to prevent obesity and non-communicable disease | Whelan 2020 | Trial protocols |
|  | Bringing alife and complex systems science to population health research | Silverman 2018 | Theoretical or commentary papers |
|  | Health by design: interweaving health promotion into environments and settings | Springer 2017 | Theoretical or commentary papers |
|  | Development and validation of an agent based model to examine neuroticism and obesity | Tanenbaum 2017 | Only testing a tool of systems thinking |
|  | School-based adolescent obesity prevention programming: perceptions of school personnel in southern appalachia | Southerland 2015 | School based |
|  | Agent-based model of peer influence on obesity | Shoham 2010 | Only testing a tool of systems thinking |
|  | Building a systems thinking prevention workforce | Bensberg 2020 | Outcomes |
|  | Developing a systems mindset in community-based prevention | Bensberg 2021 | Outcomes |
|  | Changing the food environment for obesity prevention: key gaps and future directions | Andersonsteeves 2014 | Theoretical or commentary papers |
|  | An agent-based model of income inequalities in diet in the context of residential segregation | Auchincloss 2011 | Only testing a tool of systems thinking |
|  | A system dynamics model to simulate sustainable interventions on chronic disease outcomes in an urban community | Brittin 2015 | Only testing a tool of systems thinking |
|  | Development of the good food planning tool: a food system approach to food security in indigenous australian remote communities | Brimblecombe 2015 | Developed a tool structure and implementation approach to enable community engagement |
|  | Building and sustaining community capacity to address childhood obesity: a 3-year mixed-methods case study of a community-academic advisory board | Brock 2019 | Describing capacity building experiences, process and barriers and facilitators related to partnership and sustainability through the use of community based participatory research |
|  | From individual behaviour strategies to sustainable food systems: countering the obesity and non communicable diseases epidemic in new zealand | Cammock 2020 | Theoretical or commentary papers |
|  | Systems thinking in 49 communities related to healthy eating, active living, and childhood obesity | Brennan 2015 | Using group model building as a part of an evaluation process |
|  | A community based systems diagram of obesity causes | Allender 2015 | Only testing a tool of systems thinking |
|  | Translating systems thinking into practice for community action on childhood obesity | Allender 2019 | Theoretical or commentary papers |
|  | Wayfinding the live 5-2-1-0 initiative-at the intersection between systems thinking and community-based childhood obesity prevention | Amed 2016 | Describing how to facilitate and sustain the beginnings of a systems-level/community-level change |
|  | Agent-based modeling of policies to improve urban food access for low-income populations | Widener 2013 | Theoretical or commentary papers |
|  | Actearly: a city collaboratory approach to early promotion of good health and wellbeing | Wright 2019 | Developed their logic model without using systems science tools or bounding the system. |
|  | A multivalued agent-based model for the study of noncommunicable diseases | Aziza 2019 | Theoretical or commentary papers |
|  | Introduction to the theme issue on dynamics of health behavior: revisiting systems science for population health | Burke 2020 | Theoretical or commentary papers |
|  | Systems science: a tool for understanding obesity | Bures 2014 | Theoretical or commentary papers |
|  | Implementing group model building with the shape up under 5 community committee working to prevent early childhood obesity in somerville, massachusetts | Calancie 2020 | Only testing a tool of systems thinking |
|  | Applications of social network analysis to obesity: a systematic review | Zhang 2018 | Review papers |
|  | San diego healthy weight collaborative: a systems approach to address childhood obesity | Serpas 2013 | did not bound the system, did not use mental models |
|  | Agent-based modeling insights into the optimal distribution of the fresh fruit and vegetable program | Schauder 2020 | Only testing a tool of systems thinking |
|  | Simulation modeling to assist with childhood obesity control: perceptions of baltimore city policymakers | Seifu 2018 | Outcomes |
|  | Projected impact of mexico's sugar-sweetened beverage tax policy on diabetes and cardiovascular disease: a modeling study | Sanchez-romero 2016 | Only testing a tool of systems thinking |
|  | Cost-effectiveness of bariatric surgical procedures for the treatment of severe obesity | Wang 2014 | Outcomes |
|  | Evaluating social network-based weight loss interventions in chinese population: an agent-based simulation | Shi 2020 | Only testing a tool of systems thinking |
|  | Using social network analysis to clarify the role of obesity in selection of adolescent friends | Schaefer 2014 | Only testing a tool of systems thinking |
|  | Investigating system-level drivers of obesity with adolescents: a group model-building exercise | Savona 2019 | Only testing a tool of systems thinking |
|  | Network interventions on physical activity in an afterschool program: an agent-based social network study | Zhang 2015 | Only testing a tool of systems thinking |
|  | Using agent-based modeling to study multiple risk factors and multiple health outcomes at multiple levels | Yang 2017 | Only testing a tool of systems thinking |
|  | Social network analysis of interdisciplinarity in obesity research | Bales 2008 | Theoretical or commentary papers |
|  | Using the behaviour change wheel for designing an online platform for healthy weight loss - "poemas" | Beleigoli 2018 | Review papers |
|  | Group model building: a framework for organizing healthy community program and policy initiatives in columbia, missouri | Thomas 2015 | Only testing a tool of systems thinking |
|  | Lost in translation? Theory, policy and practice in systems-based environmental approaches to obesity prevention in the healthy towns programme in england | Sautkina 2014 | Outcomes |
|  | Estimating the household drought driven food insecurity using system dynamics model: the case of afar national regional state of ethiopia | Belay 2019 | Only testing a tool of systems thinking |
|  | Whole systems approaches to obesity and other complex public health challenges: a systematic review | Bagnall 2019 | Review papers |
|  | Comparing methods of targeting obesity interventions in populations: an agent-based simulation | Beheshti 2017 | Only testing a tool of systems thinking |
|  | Applications of systems modelling in obesity research | Xue 2018 | Review papers |
|  | Leveraging social influence to address overweight and obesity using agent-based models: the role of adolescent social networks | Zhang 2015 | Only testing a tool of systems thinking |
|  | Agent-based modeling of social norm impacts on obesity and eating behaviors among school children in china and the united states | Xue 2017 | Only testing a tool of systems thinking |
|  | The interaction of social networks and child obesity prevention program effects: the pathways trial | Shin 2014 | Only testing a tool of systems thinking |
|  | A systems thinking methodology for studying prevention efforts in communities | Riley, t 2021 | Theoretical or commentary papers |
|  | A system-level approach to overweight and obesity in the veterans health administration | Raffa 2017 | Theoretical or commentary papers |
|  | Network effects on adolescents' perceived barriers to physical activity | Prochnow 2020 | Only testing a tool of systems thinking |
|  | Social network analysis in child and adolescent physical activity research: a systematic literature review | Prochnow 2020 | Review papers |
|  | 'Nothing can be done until everything is done': the use of complexity arguments by food, beverage, alcohol and gambling industries | Petticrew 2017 | Theoretical or commentary papers |
|  | Neighbourhood food, physical activity, and educational environments and black/white disparities in obesity: a complex systems simulation analysis | Orr 2016 | Only testing a tool of systems thinking |
|  | Integrating mhealth and systems science: a combination approach to prevent and treat chronic health conditions | Oreskovic 2015 | Theoretical or commentary papers |
|  | Application of virtual reality methods to obesity prevention and management research | Persky 2011 | Theoretical or commentary papers |
|  | Approaching the wicked problem of obesity: an introduction to the food system compass | Parkinson 2017 | Theoretical or commentary papers |
|  | An exploration using system dynamics modelling of population-level mindfulness, mindful eating and healthy weight following intervention | Roesler 2020 | Only testing a tool of systems thinking |
|  | Can the target set for reducing childhood overweight and obesity be met? A system dynamics modelling study in new south wales, australia | Roberts 2019 | Only testing a tool of systems thinking |
|  | The application of systems science to addressing obesity at the workplace: tapping into unexplored potential | Pronk 2016 | Theoretical or commentary papers |
|  | Systems thinking and simulation modeling to inform childhood obesity policy and practice | Powell 2017 | Theoretical or commentary papers |
|  | Whole of systems approaches to physical activity policy and practice in australia: the asapa project overview and initial systems map | Bellew 2020 | Using PA systems maps for advancing systems approach applications |
|  | Projected impact of a reduction in sugar-sweetened beverage consumption on diabetes and cardiovascular disease in argentina: a modeling study | Salgado 2020 | Outcomes |
|  | Together stronger: boundary work within an australian systems-based prevention initiative | Roussy 2020 | Theoretical or commentary papers |
|  | An economic assessment of analogue basal-bolus insulin versus human basal-bolus insulin in subjects with type 1 diabetes in the uk | Palmer 2007 | Outcomes |
|  | Understanding a successful obesity prevention initiative in children under 5 from a systems perspective | Owen 2018 | Only testing a tool of systems thinking |
|  | Community priority index: utility, applicability and validation for priority setting in community-based participatory research | Salihu 2015 | Only testing a tool of systems thinking |
|  | A systems analysis of health enhancing physical activity policy making at local level in romania | Sandu 2015 | Conference papers |
|  | Engaging community stakeholders for school-based physical activity intervention | Salsberg 2015 | Didn’t use mental models and school based (not a population level) |
|  | Developing a systems-based framework of the factors influencing dietary and physical activity behaviours in ethnic minority populations living in europe - a dedipac study | Holdsworth 2017 | Only testing a tool of systems thinking |
|  | Behavior-over-time graphs: assessing perceived trends in healthy eating and active living environments and behaviors across 49 communities | Hoehner 2015 | Only testing a tool of systems thinking |
|  | Addressing childhood obesity at every well-child visit - a systems approach to incorporating screening, prevention, and treatment into the primary care office visit | Kleven 2018 | Theoretical or commentary papers |
|  | Healthy kids, healthy cuba: findings from a group model building process in the rural southwest | Keane 2015 | Only testing a tool of systems thinking |
|  | Activating a community: an agent-based model of romp & chomp, a whole-of-community childhood obesity intervention | Kasman 2019 | Only testing a tool of systems thinking |
|  | Social networks and obesity among somali immigrants and refugees | Njeru 2020 | Only testing a tool of systems thinking |
|  | Agent-based modeling of noncommunicable diseases: a systematic review | Nianogo 2015 | Review papers |
|  | Collaboration in complex systems: multilevel network analysis for community-based obesity prevention interventions | Mcglashan 2019 | Only testing a tool of systems thinking |
|  | Systems science methods in public health: dynamics, networks, and agents | Luke 2012 | Review papers |
|  | Current situation and progress toward the 2030 health-related sustainable development goals in china: a systematic analysis | Chen 2019 | Review papers |
|  | Diet and physical activity of korean female adolescents in their peer networks | Chung 2019 | Only testing a tool of systems thinking |
|  | Understanding health promotion policy processes: a study of the government adoption of the achievement program in victoria, australia | Clarke 2018 | Only testing a tool of systems thinking |
|  | Environmental components of childhood obesity prevention interventions: an overview of systematic reviews | Cauchi 2016 | Review papers |
|  | Using system mapping to help plan and implement city-wide action to promote physical activity | Cavill 2020 | Only testing a tool of systems thinking |
|  | Systems science and systems thinking for public health: a systematic review of the field | Carey 2015 | Review papers |
|  | Next steps in obesity prevention: applying the systems approach | Huang 2013 | Theoretical or commentary papers |
|  | A systematic review of the evaluation of interventions to tackle children's food insecurity | Holley 2019 | Review papers |
|  | Examining disparities in food accessibility among households in columbus, ohio: an agent-based model | Koh 2019 | Only testing a tool of systems thinking |
|  | Designing an agent-based model for childhood obesity interventions: a case study of childobesity180 | Hennessy 2016 | Only testing a tool of systems thinking |
|  | The secure model: a comprehensive approach for obesity management | Kapoor 2020 | Theoretical or commentary papers |
|  | Systems science and obesity policy: a novel framework for analyzing and rethinking population-level planning | Johnston 2014 | Review papers |
|  | Complex systems approaches to diet: a systematic review | Langellier 2019 | Review papers |
|  | An investigation of factors affecting elementary school students' bmi values based on the system dynamics modeling | Lan 2014 | Only testing a tool of systems thinking |
|  | Simulating the impact of sugar-sweetened beverage warning labels in three cities | Lee 2018 | Only testing a tool of systems thinking |
|  | A systems approach to obesity | Lee 2017 | Theoretical or commentary papers |
|  | School-based systems change for obesity prevention in adolescents: outcomes of the australian capital territory 'it's your move!' | Malakellis 2017 | School based, didn’t use mental models |
|  | Systems science for obesity-related research questions: an introduction to the theme issue | Mabry 2014 | Theoretical or commentary papers |
|  | System dynamics modeling of childhood obesity | Madahian 2011 | Only testing a tool of systems thinking |
|  | Evidence, theory and context--using intervention mapping to develop a school-based intervention to prevent obesity in children | Lloyd 2011 | School based, didn’t use mental models |
|  | Assessing lifestyle interventions to improve cardiovascular health using an agent-based model | Li 2014 | Only testing a tool of systems thinking |
|  | Agent-based modeling of chronic diseases: a narrative review and future research directions | Li 2016 | Review papers |
|  | Understanding out-of-home food environment, family restaurant choices, and childhood obesity with an agent-based huff model | Li 2018 | Only testing a tool of systems thinking |
|  | Simulation models of obesity: a review of the literature and implications for research and policy | Levy 2011 | Review papers |
|  | Social network analysis of group position, popularity, and sleep behaviors among us adolescents | Li 2019 | Only testing a tool of systems thinking |
|  | Use of group model building to develop implementation strategies for early childhood obesity prevention | Korn 2017 | Only testing a tool of systems thinking |
|  | Comparing complex perspectives on obesity drivers: action-driven communities and evidence-oriented experts | Mcglashan 2018 | Theoretical or commentary papers |
|  | Using a system dynamics model to study the obesity transition by socioeconomic status in colombia at the country, regional and department levels | Meisel 2020 | Only testing a tool of systems thinking |
|  | Social network analysis of stakeholder networks from two community-based obesity prevention interventions | Mcglashan 2018 | Only testing a tool of systems thinking |
|  | Evaluating a community-based public health intervention using a complex systems approach | Matheson 2018 | Theoretical or commentary papers |
|  | Networks for prevention in 19 communities at the start of a large-scale community-based obesity prevention initiative | Marks 2018 | Only testing a tool of systems thinking |
|  | A systematic approach to evaluating public health training: the obesity prevention in public health course | Mainor 2014 | Theoretical or commentary papers |
|  | Neighbourhood effects on body constitution-a case study of hong kong | Low 2016 | Only testing a tool of systems thinking |
|  | Systems simulation model for assessing the sustainability and synergistic impacts of sugar-sweetened beverages tax and revenue recycling on childhood obesity prevention | Liu 2016 | Only testing a tool of systems thinking |
|  | Applying pragmatic approaches to complex program evaluation: a case study of implementation of the new south wales get healthy at work program | Crane 2019 | Theoretical or commentary papers |
|  | Understanding the livelighter® obesity prevention policy processes: an investigation using political science and systems thinking | Clarke 2020 | Theoretical or commentary papers |
|  | Understanding how local authorities in england address obesity: a wider determinants of health perspective | Nobles 2019 | Theoretical or commentary papers |
|  | A system dynamics model of the nutritional stages of the colombian population | Meisel 2016 | Only testing a tool of systems thinking |
|  | A systematic approach for the development of a kindergarten-based intervention for the prevention of obesity in preschool age children: the toybox-study | Manios 2012 | School based, didn’t use mental models |
|  | Using social network analysis to identify key child care center staff for obesity prevention interventions: a pilot study | Marks 2013 | Only testing a tool of systems thinking |
|  | Investigating the diffusion of agent-based modelling and system dynamics modelling in population health and healthcare research | Liu 2018 | Review papers |
|  | Integrating models of human behaviour between the individual and population levels to inform conservation interventions | Dobson 2019 | Theoretical or commentary papers |
|  | Systems science and childhood obesity: a systematic review and new directions | Cockrellskinner 2013 | Review papers |
|  | School beverage environment and children's energy expenditure associated with physical education class: an agent-based model simulation | Chen 2017 | Only testing a tool of systems thinking |
|  | Applying systems thinking to knowledge mobilisation in public health | Haynes 2020 | Theoretical or commentary papers |
|  | Changing the future of obesity: science, policy, and action | Gortmaker 2011 | Theoretical or commentary papers |
|  | Incorporating systems science principles into the development of obesity prevention interventions: principles, benefits, and challenges | Gittelsohn 2015 | Theoretical or commentary papers |
|  | Impact of a community-based pilot intervention to tackle childhood obesity: a 'whole-system approach' case study | Gadsby 2020 | Didn’t bound the system, did not use mental models. |
|  | Cost-effectiveness of preventing weight gain and obesity: what we know and what we need to know | Gandjour 2012 | Theoretical or commentary papers |
|  | Using agent-based models to develop public policy about food behaviours: future directions and recommendations | Giabbanelli 2017 | Theoretical or commentary papers |
|  | Using systems science to understand the determinants of inequities in healthy eating | Friel 2017 | Theoretical or commentary papers |
|  | Using group model building to describe the system driving unhealthy eating and identify intervention points: a participatory, stakeholder engagement approach in the caribbean | Guariguata 2020 | Only testing a tool of systems thinking |
|  | Health warnings on sugar-sweetened beverages: simulation of impacts on diet and obesity among u.s. Adults | Grummon 2019 | Only testing a tool of systems thinking |
|  | Changing the future of obesity: science, policy, and action editorial comment | Gortmaker 2012 | Theoretical or commentary papers |
|  | Tools and analytic techniques to synthesise community knowledge in cbpr using computer-mediated participatory system modelling | Hayward 2020 | Only testing a tool of systems thinking |
|  | Tackling poverty, treating obesity: a 'whole system' approach | Hayre 2020 | Theoretical or commentary papers |
|  | A community-based system dynamics approach suggests solutions for improving healthy food access in a low-income urban environment | Mui 2019 | Outcomes |
|  | A systems science perspective and transdisciplinary models for food and nutrition security | Hammond 2012 | Theoretical or commentary papers |
|  | Integrating complex systems thinking into epidemiologic research | Naimi 2016 | Theoretical or commentary papers |
|  | Using asset mapping to engage youth in community-based participatory research: the we project | Mosavel 2018 | Only testing a tool of systems thinking |
|  | Development of a systems science curriculum to engage rural african american teens in understanding and addressing childhood obesity prevention | Frerichs 2018 | Only testing a tool of systems thinking |
|  | Applying a mixed-methods evaluation to healthy kids, healthy communities | Brownson 2015 | Approach was not applied beyond the intervention development stage |
|  | Advancing systems thinking through the healthy kids, healthy communities evaluation | Sallis 2015 | Approach was not applied beyond the intervention development stage |
|  | B‘More Healthy Communities for Kids (BHCK) | Gittelsohn 2014 | Approach was not applied beyond the intervention development stage |
|  | Intervening to reduce obesity: an agent-based modeling approach to assess the efficacy of network-based interventions | El-sayed 2012 | Only testing a tool of systems thinking |
|  | Promoting health at the community level: thinking globally, acting locally | Economos 2012 | Theoretical or commentary papers |
|  | Systems mapping of unhealthy food environments in auckland schools: a case study | D'souza 2017 | Only testing a tool of systems thinking |
|  | Using group model building to understand factors that influence childhood obesity in an urban environment | Nelson 2015 | Only testing a tool of systems thinking |
|  | The importance of systems thinking to address obesity | Finegood 2012 | Theoretical or commentary papers |
|  | Three decades of new zealand adults obesity trends: an estimation of energy imbalance gaps using system dynamics modeling | Fallah-fini 2019 | Only testing a tool of systems thinking |
|  | A systematic review of system dynamics and agent-based obesity models: evaluating obesity as part of the global syndemic | Morshed 2019 | Review papers |
|  | A multifactorial obesity model developed from nationwide public health exposome data and modern computational analyses | Gittner 2017 | Only testing a tool of systems thinking |
|  | Social network analysis of obesity-related behaviors in african american church-based friendships | Nam 2019 | Only testing a tool of systems thinking |
|  | Mind maps and network analysis to evaluate conceptualization of complex issues: a case example evaluating systems science workshops for childhood obesity prevention | Frerichs 2018 | Only testing a tool of systems thinking |
|  | A scoping review of simulation modeling in built environment and physical activity research: current status, gaps, and future directions for improving translation | Frerichs 2019 | Review papers |
|  | Exploring network structure and the role of key stakeholders to understand the obesity prevention system in an australian metropolitan health service: study protocol | Jancey 2019 | Trial protocols |
|  | Social determinants of health inequalities: towards a theoretical perspective using systems science | Jayasinghe 2015 | Theoretical or commentary papers |
|  | Addressing obesity in Stevenage, Hertfordshire: a consultation with young people | Hamilton 2019 | Didn’t use mental model |
|  | Sheffield-let's change4life: a whole systems approach to tackling overweight and obesity in children, young people and families-a local evaluation report | Copeland 2020 | Evaluation report for local efforts |
|  | Process evaluation and lessons learned from engaging local policymakers in the b'more healthy communities for kids trial | Nam 2019 | Not reporting behavioural or anthropometric outcomes |
|  | Campbelltown - changing our future: study protocol for a whole of system approach to childhood obesity in south western sydney | Maitland 2019 | Trial protocols |

**Supplementary 3: The quality assessment of the included SW-CRT**

| **Items** | | **Required Information to Meet Criteria** | **Reviewer’s Judge** |
| --- | --- | --- | --- |
| Title | | Identification of the study as stepped wedge trial | Yes |
| Authors | | Contact details for the corresponding author | Yes |
| Trial design | | Description of the trial design (e.g., parallel, cluster, non-inferiority, stepped wedge) | Yes |
| Methods | Participants | Eligibility criteria for participants/clusters and the settings where the data were  collected | Yes |
|  | Interventions | Interventions intended for each group (cluster) | Yes |
|  | Objective | Specific objective or hypothesis | Yes |
|  | Outcome | Clearly defined primary outcome for this report | Yes |
|  | Randomisation | How participants/clusters were allocated to interventions | No |
|  | Blinding | Whether or not participants, caregivers, and those assessing the outcomes were  blinded to group assignment | NA |
| Results | Numbers randomised | Number of clusters (number of participants) randomised to each group; | Yes |
|  | Recruitment | Trial status | No |
|  | Number analysed | Number of participants analysed in each group (cluster) | Yes |
|  | Outcome | For the primary outcome, a result for each group (cluster) and the estimated effect  size and its precision | Yes |
|  | Harms | Important adverse events or side effects | Yes |
|  | Conclusions | General interpretation of the results | Yes |
| Trial registration | | Registration number and name of trial register | Yes |
| Funding | | Source of funding | No |

**Supplementary** **4: The quality assessment of the included process evaluation**

| **Study details** | **Characteristics of process evaluation** | |
| --- | --- | --- |
| Stated aim of study | To examine the SEA Change Portland process to identify significant events, enablers and barriers of its development and implementation to date | |
| Methods | Data collection: Semi-structured interviews  Data analysis: Data were analysed under three key themes: collective impact, systems thinking, and asset-based community development (ABCD) | |
| Details of participants | Eight steering group members and three community task team members. | |
| Details of the programme | Description: community-led obesity prevention initiative  Target population: children aged 7–12 years  Theory: a systems-based approach  Setting: The regional seaside town of Portland  Length/intensity: 12 months | |
| **Quality appraisal** | | |
| **Questions** | **Reviewer judgment** | **Description** |
| Were steps taken to minimise bias and error/increase rigour in sampling? | Cannot tell | Limited description of sampling method |
| Were steps taken to minimise bias and error/increase rigour in data collection? | Yes | Semi-structured, participants given the opportunity to ask questions and open-ended questions used, follow-up questions |
| Were steps taken to minimise bias and error/increase rigour in data analysis? | Cannot tell | Thematic analysis conducted by one  Interviewer, Insufficient detail to judge |
| Were the findings of the study grounded in/supported by data? | Yes | The data presented illuminate/illustrate the findings |
| There was good breadth and/or depth achieved in the findings? | Yes | Good breadth and depth |
| The perspectives of interviewees were privileged? | Yes | Their ‘voice’ is clearly represented in the data. |
| **Overall reliability and usefulness of findings** | | |
| Reliability of findings | High | |
| Usefulness of findings | High | |
